# Metabolic and molecular regulation in skeletal muscle dysfunction and regeneration

**DOI:** 10.3389/fcell.2025.1651553

**Published:** 2025-08-14

**Authors:** Jong Beom Jin, Angelique Robinson, Tori Soukup, Ember Black, André Abit, Shane M. Hammer, Anna Han, Edralin Lucas, Yoo Kim, Jiyoung Bae

**Affiliations:** ^1^ Department of Nutritional Sciences, Oklahoma State University, Stillwater, OK, United States; ^2^ University of Oklahoma College of Medicine, Oklahoma Health Science Center, Oklahoma City, OK, United States; ^3^ School of Kinesiology, Applied Health, and Recreation, Oklahoma State University, Stillwater, OK, United States; ^4^ Department of Food Science and Human Nutrition, Jeonbuk National University, Jeonju, Jeollabuk-do, Republic of Korea; ^5^ K-Food Research Center, Jeonbuk National University, Jeonju, Jeollabuk-do, Republic of Korea

**Keywords:** skeletal muscle, regeneration, metabolism, mechanism, metabolic diseases

## Abstract

Skeletal muscle is an important organ in the human body for maintaining overall strength and mobility. Skeletal muscle has the capability of self-regeneration, which can be achieved by utilizing specific energy pathways. Therefore, understanding the energy metabolism of skeletal muscle is essential to exploring its regenerative mechanisms. This review addresses the current progress in understanding the essential role of metabolic pathways in skeletal muscle function, regeneration, and muscle dysfunction as it relates to diseases such as type 2 diabetes mellitus (T2DM), obesity, and aging (sarcopenia). Furthermore, we explore the fundamental metabolisms of skeletal muscle while considering not only disease progression but also therapeutic strategies. Experimental models (*in vivo* and *in vitro*) and other signaling pathways are additionally discussed while proposing that the association between energy metabolism markers and metabolic diseases in skeletal muscle could provide innovative implications. Finally, the need for developing human-relevant models to study muscle regeneration is emphasized as most current findings are derived from *in vivo* and *in vitro* models.

## Introduction

Skeletal muscle is essential for maintaining strength and mobility, with an ability to regenerate after injury (Kangalgil et al., 2024). However, due to the accumulation of stress resulting from continuous over-exercising, illnesses, injuries, or aging, skeletal muscle becomes susceptible to significant muscle mass and strength loss (sarcopenia), reduced regenerative ability, and weakened physical performance (Distefano and Goodpaster, 2018). Skeletal muscle regeneration is crucial for preserving muscle function, as it plays an important role in mobility, strength, and overall physical performance (Ahmad et al., 2023). Muscles can continuously lose their function when regenerative processes are impaired (Ahmad et al., 2023). Reduced regenerative capabilities of skeletal muscle can lead to chronic diseases such as atrophy and fibrosis (Muscaritoli et al., 2013). With aging, the capacity for muscle regeneration declines, leading to an increased risk of muscle-related diseases such as sarcopenia (Muscaritoli et al., 2013). Therefore, maintaining skeletal muscle regeneration is crucial for physical health and the prevention of chronic diseases (Alway et al., 2023). Understanding the mechanisms of skeletal muscle regeneration is essential for developing robust therapeutic strategies to enhance muscle regeneration (Alway et al., 2023).

Metabolism involves a series of cellular reactions essential for preserving life by providing energy for cellular functions, and these cellular reactions occur in the cytosol and mitochondria, primarily utilizing glucose or fatty acids as energy sources (Judge and Dodd, 2020). Understanding the processes of energy production and consumption is essential for investigating mechanisms during skeletal muscle regeneration (Judge and Dodd, 2020). Skeletal muscle generates energy by breaking down carbohydrates, fats, and proteins (Hargreaves and Spriet, 2020). Glycolysis in the cytoplasm produces a small amount of ATP, and pyruvate from glycolysis enters the mitochondria, where it undergoes the tricarboxylic acid cycle (TCA) and electron transport chain (ETC.) to produce most of the ATP required for cellular function (Hargreaves and Spriet, 2020). Through these processes, mitochondria regulate skeletal muscle metabolism and adapt to conditions such as muscle disuse, aging, and disease (Hargreaves and Spriet, 2020). Mitochondrial function and structure play a key role in maintaining and stabilizing muscle energy reserve (Hood et al., 2019).

**FIGURE 1:**
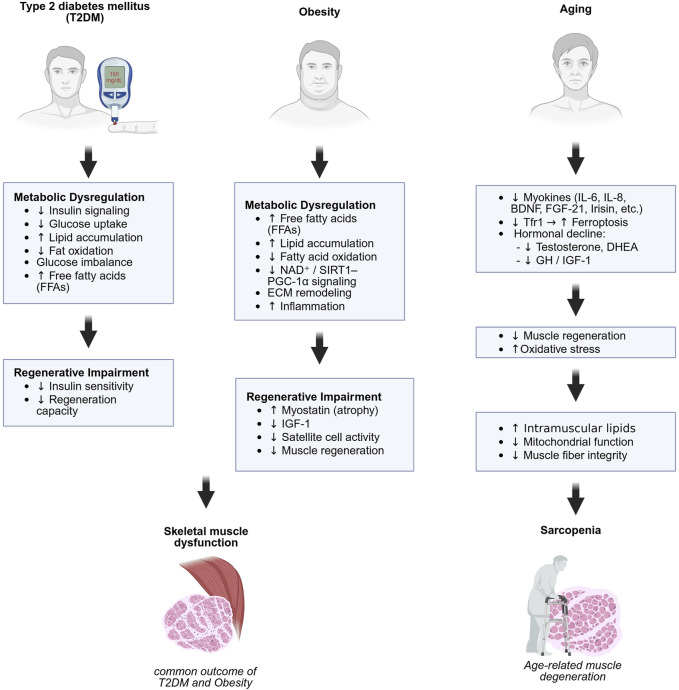
Metabolic and regenerative mechanisms underlying skeletal muscle dysfunction and sarcopenia. Distinct but overlapping mechanisms contribute to skeletal muscle impairment in type 2 diabetes (left), obesity (center), and aging (right). Type 2 diabetes and obesity involve metabolic dysregulation and impaired regeneration, leading to muscle dysfunction. Aging contributes to sarcopenia through hormonal decline, oxidative stress, and impaired mitochondrial and regenerative function. Created in BioRender.

**FIGURE 2:**
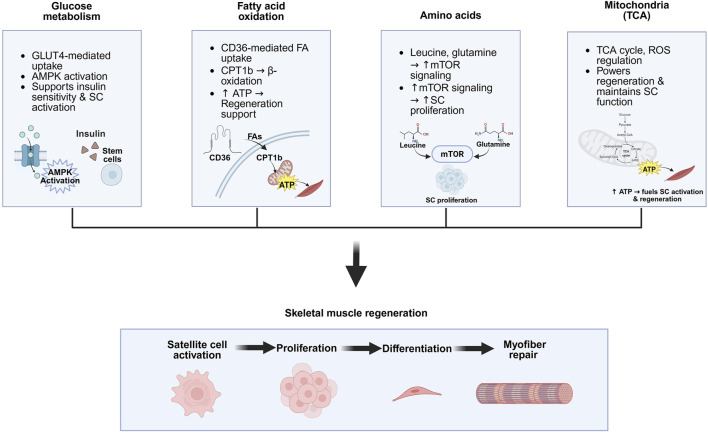
Key metabolic pathways involved in skeletal muscle regeneration. Glucose metabolism supports muscle regeneration by enhancing insulin sensitivity, GLUT4-mediated uptake, and AMPK activation, which promotes satellite cell (SC) activity. Lipid metabolism contributes via CD36-mediated fatty acid uptake and CPT1b-driven β-oxidation, generating ATP that supports regenerative processes. Amino acids such as leucine and glutamine activate mTOR signaling, which promotes SC proliferation. Mitochondrial metabolism, through the TCA cycle, supplies ATP for regeneration and regulates ROS levels, helping maintain SC function and muscle integrity. Created in BioRender.

This review aims to provide an overview of the current understanding of the essential role of metabolism in skeletal muscle function and regeneration, with a focus on how metabolic diseases such as type 2 diabetes mellitus (T2DM), obesity, and aging-related sarcopenia contribute to muscle dysfunction (Figure 1). We also discuss key experimental models and metabolic signaling pathways that regulate skeletal muscle regeneration (Figure 2). Understanding the fundamental mechanisms of skeletal muscle regeneration provides deeper insights into muscle regeneration, disease progression, and potential therapeutic strategies.

## Metabolic diseases and muscle dysfunction

### Type 2 diabetes (T2DM)

Skeletal muscle is the major site of glucose uptake in the postprandial state in humans. Under euglycemic hyperinsulinemic conditions, approximately 80% of glucose uptake occurs in skeletal muscle (DeFronzo and Tripathy, 2009). Skeletal muscle insulin resistance, a hallmark of T2DM, caused by a reduced response to insulin, impairs glucose uptake and disrupts the maintenance of normal glucose levels after food intake (DeFronzo and Tripathy, 2009).

Mitochondrial dysfunction plays an important role in the development of T2DM and contributes to subsequent skeletal muscle dysfunction (Phielix and Mensink, 2008). In T2DM, skeletal muscle dysfunction is characterized by elevated levels of circulating free fatty acids (FFAs), which impair fat oxidation and promote lipid accumulation in muscle cells and lead to reduced insulin-stimulated glucose uptake (Phielix and Mensink, 2008). Mitochondrial dysfunction in skeletal muscle contributes to acute insulin resistance by reducing mitochondrial function and increasing oxidative stress (Lee et al., 2021). Skeletal muscle from C57BL/6 mice fed a high-fat diet to induce acute or chronic insulin resistance shows significant mitochondrial dysfunction, as evidenced by reduced citrate synthase activity, ATP production, mitochondrial DNA content, and oxygen consumption (Lee et al., 2021).

Currently, there are no FDA-approved drugs specifically targeting skeletal muscle dysfunction in metabolic diseases. In contrast, pharmacological regimens for inherited muscular disorders, such as Duchenne muscular dystrophy (e.g., Eteplirsen, Casimersen), and for acute or chronic musculoskeletal pain (e.g., Carisoprodol, Diazepam) are actively used in clinical practice. Interestingly, several established medications for T2DM have positively influenced skeletal muscle function. Tofogliflozin, a novel sodium glucose co-transporter 2 (SGLT2) inhibitor, improves metabolic outcomes, including insulin resistance in skeletal muscle and lipolysis in adipose tissue (Obata et al., 2016). A recent clinical study further demonstrated that Tofogliflozin enhances glucose metabolism and insulin sensitivity without inducing muscle loss in patients with non-alcoholic fatty liver disease (Goya et al., 2022). Additionally, the endothelial DII4-muscular Notch2 signaling axis has been identified as a key regulator of skeletal muscle mass under diabetic conditions, offering a novel therapeutic target for maintaining muscle homeostasis in T2DM (Fujimaki et al., 2022).

In contrast, chronic use of metformin, the first-line treatment for T2DM, has been linked to muscle atrophy in some patients (Das et al., 2012; de Fatima Silva et al., 2018; Baker et al., 2021). Metformin has been shown to downregulate myostatin expression via the AMPK-FoxO3a-HDAC signaling pathway (Kang et al., 2022). This mechanism suggests that careful monitoring may be necessary to prevent potential muscle loss in patients with T2DM.

### Obesity

Obesity impairs skeletal muscle function by decreasing mobility, strength, and balance and is also linked to impaired skeletal muscle regeneration and function due to interrupted muscle metabolic processes, including insulin resistance, increased fatty acid absorption, and intramuscular fat accumulation (Tomlinson et al., 2016; Verpoorten et al., 2020).

Lipid overfeeding in patients with obesity induces a metabolic shift in skeletal muscle, characterized by increased lipid accumulation, reduced fatty acid oxidation, and downregulation of PDK4, potentially due to inhibition of the SIRT1-PGC-1α pathway and reduced NAD^+^ levels (Seyssel et al., 2014). Impaired phospholipid methylation in skeletal muscle has been linked to reduced calcium transport efficiency, leading to decreased energy expenditure and contributing to obesity-related metabolic dysfunction (Verkerke et al., 2019). Obesity also alters extracellular matrix (ECM) organization in both lung and muscle tissues post-injury, leading to decreased expression of ECM-related genes, impaired muscle force recovery, reduced satellite cell activation, and prolonged inflammatory responses (Gihring et al., 2020). In sarcopenic obesity, skeletal muscle maintenance and regenerative potential are also severely compromised due to altered cell cycle progression, inflammatory signaling, and disrupted protein turnover due to upregulation of MyoD and downregulation of IGF-1 gene expression (Brown et al., 2021). A high-fat diet also altered fatty acid composition, such as saturated fatty acids (SFA), monounsaturated fatty acids (MUFA), and polyunsaturated fatty acids (PUFA) in both phospholipid and triglyceride fractions in the skeletal muscle (Werner et al., 2018).

Several therapeutic strategies targeting obesity have demonstrated efficacy in modulating skeletal muscle function. Celastrol treatment mitigates obesity-induced metabolic dysfunctions by enhancing insulin sensitivity in skeletal muscle via upregulation of GLUT4, improvement of mitochondrial function, and activation of antioxidant defense mechanisms (Abu Bakar et al., 2020). Metformin also reduces fat mass, mitigates ectopic lipid accumulation, and lowers systemic inflammation markers in both sarcopenic and obese sarcopenic mice, potentially via AMPK activation (Rowan et al., 2018). Interestingly, unlike its effects in T2DM, metformin treatment may play a beneficial role in skeletal muscle function in individuals with obesity. Maternal metformin treatment improves impaired skeletal muscle development in offspring exposed to an obesogenic environment (Rowan et al., 2018), and metformin treatment has been shown to prevent a high-fat diet induced myofiber atrophy and fibrosis via the PGC-1α/FOXO3 signaling pathways (Hasan et al., 2019). These findings suggest a critical need for further investigation into the differential effects of metformin on skeletal muscle function across various metabolic diseases.

### Aging (sarcopenia)

Sarcopenia is a progressive loss of skeletal muscle mass and intensity associated with aging and significantly affects mobility and independence in the elderly (Tosato et al., 2024). Skeletal muscle peaks in early adulthood, followed by a gradual decline that becomes more pronounced after age 40 (Tosato et al., 2024).

There are various markers associated with sarcopenia progression. Myokines are cytokines secreted by skeletal muscle, including interleukin (IL)-6, IL-8, IL-5, brain-derivded neurotrophic factor (BDNF), fibroblast growth factor (FGF)-21, leukemia hibihitory factor (LIF), irisin and secreted protein acidic and rich in cysteine (SPARC) and are responsible for inhibiting the progression of sarcopenia, but its ability to secrete decreases with age (Aryana et al., 2018). The deletion of transferrin receptor 1 (Tfr1) in skeletal muscle aging impairs skeletal muscle regeneration through the activation of ferroptosis (Ding et al., 2021). Sarcopenia is also characterized by a wide range of fluctuations in various hormones, especially sex hormones such as testosterone and dehydroepiandrosterone (DHEA), and growth hormones such as growth hormone (GH) and insulin-like growth factor 1 (IGF-1) (Curcio et al., 2016).

There are several ways to prevent sarcopenia in relation to aging. Overexpression of the antioxidant enzyme glutathione peroxidase 4 (GPX4) can reduce oxidative damage, preserve muscle mass, and maintain mitochondrial function, thereby potentially mitigating the effects of sarcopenia (Czyżowska et al., 2023). Intramyocellular lipids (IMCL) and extramyocellular lipids (EMCL) have distinct impacts on muscle and physical function, less lipid accumulation is generally associated with better performance in muscle function and activities of daily living (ADLs) in healthy, non-athlete older individuals (Yoshiko et al., 2022). Vitamin D intake improved muscle quality in elderly men and positively affected fiber type morphology and myostatin expression in young men (Agergaard et al., 2015). These results suggest that vitamin D might contribute to better muscle function and quality, particularly in older adults (Agergaard et al., 2015).

Several studies have reported a potential protective effect of metformin against sarcopenia (Lyu et al., 2022; Petrocelli et al., 2021; Long et al., 2017). Metformin significantly activates AMPK signaling pathways and suppresses the expression of pro-inflammatory cytokines in the skeletal muscle of sarcopenic mice (Lyu et al., 2022). Furthermore, metformin and leucine co-treatment has been shown to improve muscle quality during aging by increasing satellite cell content but reducing collagen accumulation in skeletal muscle (Petrocelli et al., 2021). In addition, a randomized controlled clinical trial demonstrated that 10 weeks of metformin administration significantly decreased the expression of inflammation-related genes in the skeletal muscle of older adults aged over 65 years (Long et al., 2017).

## Metabolic pathways in muscle function and regeneration

### Glucose metabolism

Skeletal muscle is a regulator of glucose homeostasis, responsible for 80% of postprandial glucose uptake from the circulation (Merz and Thurmond, 2020). It is essential for metabolism, both for its role in glucose uptake and its importance in exercise and metabolic disease (Merz and Thurmond, 2020). Skeletal muscle is the predominant site of insulin-mediated glucose uptake in the postprandial state (DeFronzo and Tripathy, 2009). Insulin resistance in skeletal muscle is particularly important since it is normally responsible for more than 75% of all insulin-mediated glucose disposal (Wei et al., 2008).

Glucose metabolism in skeletal muscle is closely associated with insulin resistance, as documented in several studies. IL-10, a Th_2_-type cytokine that is produced by a wide range of immunological cell types, increased insulin sensitivity and protects skeletal muscle from obesity-associated macrophage infiltration, increases in inflammatory cytokines, and their deleterious effects on insulin signaling and glucose metabolism (Hong et al., 2009). Secretions of tumor necrosis factor α (TNF-α) activated macrophage-induced insulin resistance in skeletal muscle in a dose-dependent manner (Bu et al., 2020). Fibroblast growth factor 21 (FGF21) increases insulin-stimulated glucose uptake in human myotubes (Mashili et al., 2011). FGF21 has direct effects in enhancing skeletal muscle glucose uptake, providing additional points of regulation that may contribute to the beneficial effects of FGF21 on glucose homeostasis (Mashili et al., 2011).

Additionally, glucose metabolism related to skeletal muscle is also involved in the process of exercise. During exercise, muscles contract and secrete factors called myokines which can act in an autocrine/paracrine fashion to improve muscle energy metabolism including glucose (Balakrishnan and Thurmond, 2022). Myokines have been shown to increase insulin sensitivity, thereby improving glucose disposal and regulating glucose and lipid metabolism (Garneau and Aguer, 2019). SPARC, an exercise-responsive myokine, improves glucose tolerance and concomitantly activates AMPK in skeletal muscle (Aoi et al., 2019).

### Fatty acid oxidation

Skeletal muscle myocyte mitochondria are primary sites of fatty acid oxidation (Watt and Hoy, 2012). Carnitine palmitoyltransferase 1 (CPT1) catalyzes the first step in long-chain fatty acid import into mitochondria, and it is believed to be rate limiting for β-oxidation of fatty acids (Sebastián et al., 2009). Plasma free fatty acids (FFAs), when entering the myocyte in skeletal muscle, are converted into fatty acyl-CoA, with the enzymatic regulation of CPT-I controlling their transport into the mitochondria for oxidation (Rasmussen and Wolfe, 1999). Once inside, β-oxidation enzymes break down fatty acyl-CoAs to generate ATP, with regulation occurring at fatty acid transport, CPT-I activity, and mitochondrial β-oxidation enzymes (Rasmussen and Wolfe, 1999). Overexpression of CPT1 in skeletal muscle is sufficient to enhance fatty acid oxidation and improve insulin resistance (Bruce et al., 2009). In skeletal muscle, carnitine palmitoyltransferase 1b (CPT1b) overexpression could be helping to maintain metabolic health with increasing age (Bétry et al., 2019).

Fatty acid oxidation is regulated in skeletal muscle during exercise, and is also determined by FA availability to mitochondria, dependent on trans-sarcolemmal FA uptake via cluster of differentiation 36/SR-B2 (CD36) and FAs mobilized from myocellular lipid droplets (Lundsgaard et al., 2018). CD36 is a scavenger receptor that functions in high affinity tissue uptake of long chain fatty acids (FA) and contributes under excessive fat supply to lipid accumulation and metabolic dysfunction (Pepino et al., 2014). CD36 functions as both a signaling receptor and fatty acid transporter in various immune and non-immune cells (Chen et al., 2022). CD36-deficient mice have impaired satellite cell differentiation and skeletal muscle regeneration, which links CD36 as a key regulator of processes involving mitochondrial fatty acid oxidation and bioenergetics (Verpoorten et al., 2020).

### Amino acids

Amino acids influence several signaling pathways that control muscle metabolism. In skeletal muscle, leucine, a nutritionally essential branched-chain amino acid (BCAA), promotes energy metabolism (glucose uptake, mitochondrial biogenesis, and fatty acid oxidation) to provide energy for protein synthesis, while inhibiting protein degradation (Nair et al., 1992; Duan et al., 2016).

Skeletal muscle is a major organ for glutamine synthesis, storage, and release (Cruzat, 2019). Low glutamine levels stimulate macrophages to increase glutamine synthase (GS) activity, reducing glutamine oxidation via glutamate dehydrogenase (GLUD)1, which ensures sufficient glutamine supply for satellite cells (Shang et al., 2020). This glutamine, taken up through solute carrier family 1 member 5 (SLC1A5), activates mammalian target of rapamycin (mTOR), promoting satellite cell proliferation and differentiation, thus enhancing muscle regeneration when GLUD1 is inhibited (Shang et al., 2020).

Extracellular metabolites such as amino acids and carbohydrates regulate muscle satellite cell function by modulating key metabolic and signaling pathways, including AMPK and mTOR. These nutrients influence satellite cell proliferation, differentiation, and overall regenerative capacity (Zhang et al., 2020; Hernandez-Benitez et al., 2024; Kang et al., 2024).

BCAAs, including isoleucine and valine, are essential for protein synthesis and play crucial roles in energy metabolism within the body. Metabolism of these BCAAs in skeletal muscle contributes to insulin resistance in humans (Lerin et al., 2016). Interruptions in BCAAs metabolic pathways can diminish cellular respiration and lipid oxidation, particularly under conditions of lipid excess, which are associated with increased weight gain, accumulation of lipids in muscle tissue, and the development of insulin resistance (Lerin et al., 2016).

### Mitochondria and the TCA cycle

The TCA cycle plays a fundamental role in energy production for skeletal muscle (Martínez-Reyes and Chandel, 2020). Mitochondria are well known as biosynthetic and bioenergy cells for their roles in generating metabolites and ATP, which are by-products of the TCA cycle and mitochondrial membrane potential, respectively (Martínez-Reyes et al., 2016). In addition, since mitochondria are important organelles that control the metabolic state of skeletal muscle, studies of mitochondrial function are important to describe the relationship between TCA cycle and skeletal muscle (Hood et al., 2019).

Alterations of skeletal muscle mitochondrial functioning can alter muscular regenerative responses (Heo et al., 2023). During exercise or metabolic stress, skeletal muscle cells utilize substrates like fatty acids, amino acids, and pyruvate, and alterations in mitochondrial function, particularly in the TCA cycle, link disruptions in energy metabolism and riboflavin biosynthesis to the pathogenesis of sarcopenia (Heo et al., 2023). The effect of altered mitochondrial functioning on skeletal muscle regenerative responses is also observed post-injury and skeletal muscle injury leads to varied TCA cycle responses (Hartmann et al., 2020).

Skeletal muscle injury affects the TCA cycle. Muscle repair is energy demanding and mitochondria provide the primary source for energy production during regeneration (Chatzinikita et al., 2023). Excessive ROS caused by skeletal muscle injury can trigger mitochondrial degradation, including mitochondrial DNA damage, electron transport chain abnormalities and disruption of the membrane potential (Lian et al., 2022). Tissue damage immediately triggers the inflammatory response (Chazaud, 2020). Particularly, in pro-inflammatory macrophages, TCA cycle intermediates (citrate, cis-Aconitate, isocitrate, α-ketoglutarate, and fumarate) are remodeled (Choi et al., 2021).

### Experimental models for studying muscle regeneration

Skeletal muscle regeneration is a highly coordinated process involving stem cell activation, immune responses, and extracellular matrix remodeling. To dissect this complexity, previous studies utilize a variety of *in vitro* and *in vivo* models, each offering distinct advantages for investigating mechanisms of myogenesis, gene regulation, and tissue regeneration under controlled or physiological conditions (Klumpp et al., 2010; Viggars et al., 2023; Ge et al., 2024).

*In vitro* models are essential tools for investigating the molecular mechanisms of skeletal muscle regeneration as they provide controlled conditions to examine satellite cell activation, myogenic differentiation, immune interactions, and pharmacological responses. Conventional two-dimensional (2D) cultures and advanced three-dimensional (3D) bioengineered muscle tissues enable the modeling of regenerative processes under various cellular microenvironments (Pillon et al., 2013; Lian et al., 2022; Chatzinikita et al., 2023). Recent developments in tissue engineering have enabled the generation of human muscle bundles that replicate clinical drug responses (Madden et al., 2015) and have led to optimized protocols for the expansion, genetic modification, and cryopreservation of primary human muscle stem cells (Juhas and Bursac, 2013). These experimental models have improved our understanding of satellite cell self-renewal, lineage specification, and regenerative signaling pathways (Zammit et al., 2004). *In vivo* models provide the physiological conditions to study muscle regeneration that involve vascularization, fibrosis, and immune cell infiltration. Rodent injury models utilizing cardiotoxin, notexin, or barium chloride (BaCl_2_) are widely used to evaluate regeneration kinetics and satellite cell behavior (Hardy et al., 2016). These models have also been used to study systemic regulators such as melatonin, which enhances mitochondrial metabolism and supports vascularized muscle regeneration (Ge et al., 2024) and intrinsic factors like the chromatin remodeler CHD4, which is essential for maintaining satellite cell identity during regeneration (Sreenivasan et al., 2021). Methodologies for modeling both acute and chronic muscle injury have been comprehensively summarized in previous reviews (Grounds et al., 2002; Viggars et al., 2023).

Alternative and emerging models are increasingly being adopted to complement conventional approaches in muscle regeneration research. Zebrafish have been used to study age-related muscle degeneration (Ichii et al., 2022) and to establish 3D muscle culture systems that mimic physiological conditions (Vishnolia et al., 2020). Bioengineered systems integrating 3D bioprinting and nanomaterials have introduced novel systems for investigating regenerative processes (Jo et al., 2024). In addition, large animal models such as dystrophic dogs, closely replicate the pathological conditions of human muscular dystrophy and serve as valuable tools for translational studies (Kornegay, 2017). Collectively, these complementary models provide experimental systems that enhance our understanding of the cellular and molecular mechanisms involved in muscle regeneration (Table 1).

**TABLE 1:** Summary of experimental models used to study skeletal muscle regeneration. Multiple *in vitro*, *in vivo*, and emerging models are employed to investigate distinct aspects of muscle regeneration. These include satellite cell activation, immune interactions, gene editing, and functional recovery. The table highlights representative examples, their research applications, and key insights derived from each system.

Model type	Specific example	Application/Purpose	Key insights/Notes
* In vitro*	2D cultures, 3D engineered tissues	SC activation, differentiation, immune interaction	High experimental control, drug testing platform
* In vitro*	Bioengineered human muscle bundles	Response to therapeutic agents	Clinically relevant responses
* In vitro*	Primary human muscle stem cells	Expansion, gene editing, cryopreservation	Optimized protocols for manipulation
* In vivo*	Rodent injury models (cardiotoxin, notexin, BaCl_2_)	Regeneration kinetics, SC behavior	Standardized and physiologically relevant
* In vivo*	Melatonin treatment	Enhanced mitochondrial metabolism and repair	Systemic modulation of muscle regeneration
* In vivo*	CHD4 chromatin remodeling	Maintenance of satellite cell identity	Highlights epigenetic regulation
Emerging	Zebrafish models	Aging muscle decline, 3D culture development	Regenerative capacity, real-time observation
Emerging	3D bioprinted constructs + nanomaterials	Advanced regeneration platforms	Mimics structure and microenvironment
Emerging	Dystrophic dogs	Modeling human muscular dystrophy	Translational large animal model

## Molecular mechanisms in muscle injury and regeneration

### Signaling pathways modulating satellite cell metabolism

Multiple signaling pathways regulate the regenerative capacity of muscle satellite cells by directing cell fate decisions and modulating key aspects of cellular metabolism. These metabolic pathways play important roles in controlling satellite cell activation, proliferation, and contribution to tissue regeneration (Forcina et al., 2020).

Satellite cell metabolism is regulated by a variety of signaling pathways that respond to mechanical forces, stress, and environmental changes. Mechanosensitive regulators such as Piezo1 modulate mitochondrial function and redox balance (Peng et al., 2022; Hirano et al., 2023), while cytoskeletal regulators like rho guanine nucleotide exchange factor 3 (ARHGEF3) promote autophagy to sustain regenerative capacity during injury or aging (You et al., 2021). Transcriptional and epigenetic factors, including neurogenic locus notch homolog (NOTCH) and DNA-methyltransferase 1 (Dnmt1), further modulate the balance between self-renewal and differentiation (Iio et al., 2021; Cariati et al., 2022; Zhou et al., 2022). Energy-sensing pathways and mitochondrial regulators also play significant roles in satellite cell activation. AMPK activation induced by stimuli such as low-intensity pulsed ultrasound enhances mitochondrial function and glucose uptake (Duan et al., 2024). Structural and membrane-associated proteins like Desmin and Tmem30a also contribute to mitochondrial stability and metabolic readiness, thereby supporting effective regenerative responses (Goldfarb and Dalakas, 2009; Winter et al., 2016; Sun et al., 2021).

Taken together, these signaling pathways regulate satellite cell metabolism through the integration of mechanical stimuli, stress responses, and intrinsic regulatory pathways. Understanding how these mechanisms modulate satellite cell fate and regenerative capacity is critical for developing targeted therapies for muscle-wasting conditions.

### Metabolic regulation of muscle satellite cell activation

Emerging evidence indicates the importance of cellular metabolism in regulating the activation and regenerative potential of muscle satellite cells. In response to systemic injury signals, quiescent satellite cells transition from a deep G_0_ state to an alert G (Alert) state and are regulated by key signaling pathways including mTORC1 and HGF/c-Met signaling (Goldfarb and Dalakas, 2009; Rodgers et al., 2014; Winter et al., 2016; Sun et al., 2021; Duan et al., 2024). Mitochondrial morphology and redox signaling through the ROS/GSH further modulate the depth of quiescence and readiness for activation (Baker et al., 2022). In addition, ketone body signaling induced by fasting has been implicated in maintaining a resilient quiescent state, suggesting that nutrient availability plays an important role in regulating muscle stem cell metabolism (Benjamin et al., 2022).

Upon activation, satellite cells undergo metabolic remodeling that influences their fate decisions. A metabolic shift from fatty acid oxidation to glycolysis reduces intracellular NAD^+^ levels and SIRT1 activity, thereby promoting differentiation through epigenetic modifications (Ryall et al., 2015). Similarly, GLUD1 deficiency leads to mitochondrial glutamate accumulation which impairs NADH shuttling and disrupts redox balance, ultimately leading to premature differentiation (Soro-Arnáiz et al., 2024). Mitochondrial dynamics also contribute to long-term regenerative capacity by maintaining energy homeostasis and regulating mitophagy during satellite cell activation (Hong et al., 2022). In addition, energy availability revealed by lipid droplet content contributes to asymmetric cell fate decisions. Cells with low lipid content tend to self-renew, whereas those with high lipid content are more likely to undergo differentiation (Yue et al., 2022). Together, these findings indicate that metabolic reprogramming is closely linked to both the activation of muscle satellite cells and the regulation of their regenerative response.

## Conclusion

In this review, we have summarized the current understanding of metabolic pathways including glucose metabolism, fatty acid oxidation, and the mitochondrial TCA cycle in relation to skeletal muscle regeneration. We also discussed key metabolic shifts, therapeutic strategies, and the relationship between skeletal muscle metabolism and physiological conditions such as obesity, T2DM, and aging-related sarcopenia. In addition, we examined various metabolic mechanisms associated with the regulation of skeletal muscle regeneration.

Although numerous studies have identified *in vivo* and *in vitro* mechanisms involved in musculoskeletal regeneration, our understanding of the comprehensive metabolic regulation of this process remains limited. Further research is needed to investigate the roles of specific genes and their regulatory elements in skeletal muscle regeneration. Moreover, human studies are essential as most current findings are derived from animal or cell-based models. To address this limitation, several research groups have begun developing human-based *in vitro* models. 3D artificial skeletal muscles have been engineered from human iPSC-derived cells, effectively recapitulating key features of muscular dystrophy (Maffioletti et al., 2018). Despite their potential, these models remain technically challenging and are not yet widely used. Thus, further development of human-relevant models will be essential for translational research in skeletal muscle regeneration.

Skeletal muscle is a major organ responsible for systemic energy metabolism. Therefore, various metabolic regulators should be investigated in relation to skeletal muscle regeneration and their associations with metabolic diseases. Skeletal muscle is adversely affected by metabolic conditions such as obesity, T2DM, and aging-related sarcopenia. Thus, further research is needed to understand how treatments targeting these diseases influence muscle regeneration. In addition, identifying metabolites that are altered during age-related muscle loss and determining whether they promote or inhibit disease progression could provide important insights into muscle maintenance and function. Overall, future studies focusing on the intersection of metabolism and skeletal muscle biology hold strong potential for new discoveries and therapeutic innovation.

## References

[B1] Abu Bakar M. H. Shariff K. A. Tan J. S. Lee L. K. (2020). Celastrol attenuates inflammatory responses in adipose tissues and improves skeletal muscle mitochondrial functions in high fat diet-induced Obese rats *via* upregulation of AMPK/SIRT1 signaling pathways. Eur. J. Pharmacol. 883, 173371. 10.1016/j.ejphar.2020.173371 32712089

[B2] Agergaard J. Trøstrup J. Uth J. Iversen J. V. Boesen A. Andersen J. L. (2015). Does vitamin-D intake during resistance training improve the skeletal muscle hypertrophic and strength response in young and elderly men? – a randomized controlled trial. Nutr. Metab. 12, 32. 10.1186/s12986-015-0029-y PMC4589960 26430465

[B3] Ahmad K. Shaikh S. Chun H. J. Ali S. Lim J. H. Ahmad S. S. (2023). Extracellular matrix: the critical contributor to skeletal muscle regeneration—a comprehensive review. Inflamm. Regen. 43, 58. 10.1186/s41232-023-00308-z 38008778 PMC10680355

[B4] Alway S. E. Paez H. G. Pitzer C. R. (2023). The role of mitochondria in mediation of skeletal muscle repair. Muscles 2, 119–163. 10.3390/muscles2020011 40757564 PMC12225497

[B5] Aoi W. Hirano N. Lassiter D. G. Björnholm M. Chibalin A. V. Sakuma K. (2019). Secreted protein acidic and rich in cysteine (SPARC) improves glucose tolerance *via* AMP-activated protein kinase activation. FASEB J. 33, 10551–10562. 10.1096/fj.201900453R 31225998

[B6] Aryana I. G. P. S. Hapsari A. A. A. R. Kuswardhani R. A. T. (2018). Myokine regulation as marker of Sarcopenia in elderly. Mol. Cell. Biomed. Sci. 2, 38. 10.21705/mcbs.v2i2.32

[B7] Baker C. Retzik-Stahr C. Singh V. Plomondon R. Anderson V. Rasouli N. (2021). Should metformin remain the first-line therapy for treatment of type 2 diabetes? Ther. Adv. Endocrinol. Metab. 12, 2042018820980225. 10.1177/2042018820980225 33489086 PMC7809522

[B8] Baker N. Wade S. Triolo M. Girgis J. Chwastek D. Larrigan S. (2022). The mitochondrial protein OPA1 regulates the quiescent state of adult muscle stem cells. Cell Stem Cell 29, 1315–1332.e9. 10.1016/j.stem.2022.07.010 35998642 PMC10249109

[B9] Balakrishnan R. Thurmond D. C. (2022). Mechanisms by which skeletal muscle myokines ameliorate insulin resistance. Int. J. Mol. Sci. 23, 4636. 10.3390/ijms23094636 35563026 PMC9102915

[B10] Benjamin D. I. Both P. Benjamin J. S. Nutter C. W. Tan J. H. Kang J. (2022). Fasting induces a highly resilient deep quiescent state in muscle stem cells *via* ketone body signaling. Cell Metab. 34, 902–918.e6. 10.1016/j.cmet.2022.04.012 35584694 PMC9177797

[B11] Bétry C. Meugnier E. Pflieger M. Grenet G. Hercberg S. Galan P. (2019). High expression of *CPT1b* in skeletal muscle in metabolically healthy older subjects. Diabetes Metab. 45, 152–159. 10.1016/j.diabet.2018.01.018 29657112

[B12] Brown L. A. Perry R. A. Haynie W. S. Lee D. E. Rosa-Caldwell M. E. Brown J. L. (2021). Moderators of skeletal muscle maintenance are compromised in sarcopenic obese mice. Mech. Ageing Dev. 194, 111404. 10.1016/j.mad.2020.111404 33249192

[B13] Bruce C. R. Hoy A. J. Turner N. Watt M. J. Allen T. L. Carpenter K. (2009). Overexpression of carnitine Palmitoyltransferase-1 in skeletal muscle is sufficient to enhance fatty acid oxidation and improve high-fat diet–induced insulin resistance. Diabetes 58, 550–558. 10.2337/db08-1078 19073774 PMC2646053

[B14] Bu L. Cao X. Zhang Z. Wu H. Guo R. Ma M. (2020). Decreased secretion of tumor necrosis factor-α attenuates macrophages-induced insulin resistance in skeletal muscle. Life Sci. 244, 117304. 10.1016/j.lfs.2020.117304 31953164

[B15] Cariati I. Scimeca M. Bonanni R. Triolo R. Naldi V. Toro G. (2022). Role of Myostatin in muscle degeneration by random positioning machine exposure: an *in vitro* Study for the treatment of Sarcopenia. Front. Physiol. 13, 782000. 10.3389/fphys.2022.782000 35185612 PMC8853288

[B16] Chatzinikita E. Maridaki M. Palikaras K. Koutsilieris M. Philippou A. (2023). The role of mitophagy in skeletal muscle damage and regeneration. Cells 12, 716. 10.3390/cells12050716 36899852 PMC10000750

[B17] Chazaud B. (2020). Inflammation and skeletal muscle regeneration: leave it to the macrophages. Trends Immunol. 41, 481–492. 10.1016/j.it.2020.04.006 32362490

[B18] Chen Y. Zhang J. Cui W. Silverstein R. L. (2022). CD36, a signaling receptor and fatty acid transporter that regulates immune cell metabolism and fate. J. Exp. Med. 219, e20211314. 10.1084/jem.20211314 35438721 PMC9022290

[B19] Choi I. Son H. Baek J.-H. (2021). Tricarboxylic acid (TCA) cycle intermediates: regulators of immune responses. Life 11, 69. 10.3390/life11010069 33477822 PMC7832849

[B20] Cruzat V. F. (2019). “Chapter 18 - glutamine and skeletal muscle,” in Nutrition and skeletal muscle. Editor Walrand S. (Academic Press), 299–313. 10.1016/B978-0-12-810422-4.00017-8

[B21] Curcio F. Ferro G. Basile C. Liguori I. Parrella P. Pirozzi F. (2016). Biomarkers in sarcopenia: a multifactorial approach. Exp. Gerontol. 85, 1–8. 10.1016/j.exger.2016.09.007 27633530

[B22] Czyżowska A. Brown J. Xu H. Sataranatarajan K. Kinter M. Tyrell V. J. (2023). Elevated phospholipid hydroperoxide glutathione peroxidase (GPX4) expression modulates oxylipin formation and inhibits age-related skeletal muscle atrophy and weakness. Redox Biol. 64, 102761. 10.1016/j.redox.2023.102761 37279604 PMC10276143

[B23] Das A. K. Yang Q.-Y. Fu X. Liang J.-F. Duarte M. S. Zhu M.-J. (2012). AMP-activated protein kinase stimulates myostatin expression in C2C12 cells. Biochem. Biophys. Res. Commun. 427, 36–40. 10.1016/j.bbrc.2012.08.138 22995402 PMC3486733

[B24] de Fatima Silva F. Ortiz-Silva M. Galia W. B. de S. Cassolla P. da Silva F. G. Graciano M. F. R. (2018). Effects of metformin on insulin resistance and metabolic disorders in tumor-bearing rats with advanced cachexia. Can. J. Physiol. Pharmacol. 96, 498–505. 10.1139/cjpp-2017-0171 29304290

[B25] DeFronzo R. A. Tripathy D. (2009). Skeletal muscle insulin resistance is the primary defect in type 2 diabetes. Diabetes Care 32, S157–S163. 10.2337/dc09-S302 19875544 PMC2811436

[B26] Ding H. Chen S. Pan X. Dai X. Pan G. Li Z. (2021). Transferrin receptor 1 ablation in satellite cells impedes skeletal muscle regeneration through activation of ferroptosis. J. Cachexia Sarcopenia Muscle 12, 746–768. 10.1002/jcsm.12700 33955709 PMC8200440

[B27] Distefano G. Goodpaster B. H. (2018). Effects of exercise and aging on skeletal muscle. Cold Spring Harb. Perspect. Med. 8, a029785. 10.1101/cshperspect.a029785 28432116 PMC5830901

[B28] Duan Y. Li F. Li Y. Tang Y. Kong X. Feng Z. (2016). The role of leucine and its metabolites in protein and energy metabolism. Amino Acids 48, 41–51. 10.1007/s00726-015-2067-1 26255285

[B29] Duan H. Chen S. Mai X. Fu L. Huang L. Xiao L. (2024). Low-intensity pulsed ultrasound (LIPUS) promotes skeletal muscle regeneration by regulating PGC-1α/AMPK/GLUT4 pathways in satellite cells/myoblasts. Cell. Signal. 117, 111097. 10.1016/j.cellsig.2024.111097 38355078

[B30] Forcina L. Cosentino M. Musarò A. (2020). Mechanisms regulating muscle regeneration: insights into the interrelated and time-dependent phases of tissue healing. Cells 9, 1297. 10.3390/cells9051297 32456017 PMC7290814

[B31] Fujimaki S. Matsumoto T. Muramatsu M. Nagahisa H. Horii N. Seko D. (2022). The endothelial Dll4–muscular Notch2 axis regulates skeletal muscle mass. Nat. Metab. 4, 180–189. 10.1038/s42255-022-00533-9 35228746

[B32] Garneau L. Aguer C. (2019). Role of myokines in the development of skeletal muscle insulin resistance and related metabolic defects in type 2 diabetes. Diabetes Metab. 45, 505–516. 10.1016/j.diabet.2019.02.006 30844447

[B33] Ge X. Wang C. Yang G. Maimaiti D. Hou M. Liu H. (2024). Enhancement of mitochondrial energy metabolism by melatonin promotes vascularized skeletal muscle regeneration in a volumetric muscle loss model. Free Radic. Biol. Med. 210, 146–157. 10.1016/j.freeradbiomed.2023.11.021 38008130

[B34] Gihring A. Gärtner F. Liu C. Hoenicka M. Wabitsch M. Knippschild U. (2020). Influence of obesity on the Organization of the Extracellular Matrix and satellite cell functions after combined muscle and thorax trauma in C57BL/6J mice. Front. Physiol. 11, 849. 10.3389/fphys.2020.00849 32848828 PMC7399228

[B35] Goldfarb L. G. Dalakas M. C. (2009). Tragedy in a heartbeat: malfunctioning desmin causes skeletal and cardiac muscle disease. J. Clin. Invest. 119, 1806–1813. 10.1172/JCI38027 19587455 PMC2701871

[B36] Goya T. Imoto K. Tashiro S. Aoyagi T. Takahashi M. Kurokawa M. (2022). The efficacy of tofogliflozin on Metabolic dysfunction-associated Fatty liver disease. Gastroenterol. Insights 13, 20–26. 10.3390/gastroent13010003

[B37] Grounds M. D. White J. D. Rosenthal N. Bogoyevitch M. A. (2002). The role of stem cells in skeletal and cardiac muscle repair. J. Histochem. Cytochem. 50, 589–610. 10.1177/002215540205000501 11967271

[B38] Hardy D. Besnard A. Latil M. Jouvion G. Briand D. Thépenier C. (2016). Comparative Study of injury models for studying muscle regeneration in mice. PLOS ONE 11, e0147198. 10.1371/journal.pone.0147198 26807982 PMC4726569

[B39] Hargreaves M. Spriet L. L. (2020). Skeletal muscle energy metabolism during exercise. Nat. Metab. 2, 817–828. 10.1038/s42255-020-0251-4 32747792

[B40] Hartmann D. D. Gonçalves D. F. Da Rosa P. C. Martins R. P. Courtes A. A. Franco J. L. (2020). A single muscle contusion promotes an immediate alteration in mitochondrial bioenergetics response in skeletal muscle fibres with different metabolism. Free Radic. Res. 54, 137–149. 10.1080/10715762.2020.1723795 32037913

[B41] Hasan M. M. Shalaby S. M. El-Gendy J. Abdelghany E. M. A. (2019). Beneficial effects of metformin on muscle atrophy induced by obesity in rats. J. Cell. Biochem. 120, 5677–5686. 10.1002/jcb.27852 30320911

[B42] Heo J. Schifino A. G. McFaline-Figueroa J. Miller D. L. Hoffman J. R. Noble E. E. (2023). Differential effects of Western diet and traumatic muscle injury on skeletal muscle metabolic regulation in male and female mice. J. Cachexia Sarcopenia Muscle 14, 2835–2850. 10.1002/jcsm.13361 37879629 PMC10751418

[B43] Hernandez-Benitez R. Wang C. Shi L. Ouchi Y. Zhong C. Hishida T. (2024). Intervention with metabolites emulating endogenous cell transitions accelerates muscle regeneration in young and aged mice. Cell Rep. Med. 5, 101449. 10.1016/j.xcrm.2024.101449 38508141 PMC10983034

[B44] Hirano K. Tsuchiya M. Shiomi A. Takabayashi S. Suzuki M. Ishikawa Y. (2023). The mechanosensitive ion channel PIEZO1 promotes satellite cell function in muscle regeneration. Life Sci. 6, e202201783. 10.26508/lsa.202201783 PMC9711862 36446523

[B45] Hong E.-G. Ko H. J. Cho Y.-R. Kim H.-J. Ma Z. Yu T. Y. (2009). Interleukin-10 prevents diet-induced insulin resistance by attenuating macrophage and cytokine response in skeletal muscle. Diabetes 58, 2525–2535. 10.2337/db08-1261 19690064 PMC2768157

[B46] Hong X. Isern J. Campanario S. Perdiguero E. Ramírez-Pardo I. Segalés J. (2022). Mitochondrial dynamics maintain muscle stem cell regenerative competence throughout adult life by regulating metabolism and mitophagy. Cell Stem Cell 29, 1298–1314.e10. 10.1016/j.stem.2022.07.009 35998641

[B47] Hood D. A. Memme J. M. Oliveira A. N. Triolo M. (2019). Maintenance of skeletal muscle Mitochondria in health, exercise, and aging. Annu. Rev. Physiol. 81, 19–41. 10.1146/annurev-physiol-020518-114310 30216742

[B48] Ichii S. Matsuoka I. Okazaki F. Shimada Y. (2022). Zebrafish models for skeletal muscle senescence: lessons from cell cultures and rodent models. Molecules 27, 8625. 10.3390/molecules27238625 36500717 PMC9739860

[B49] Iio H. Kikugawa T. Sawada Y. Sakai H. Yoshida S. Yanagihara Y. (2021). DNA maintenance methylation enzyme Dnmt1 in satellite cells is essential for muscle regeneration. Biochem. Biophys. Res. Commun. 534, 79–85. 10.1016/j.bbrc.2020.11.116 33310192

[B50] Jo H. J. Kang M. S. Heo H. J. Jang H. J. Park R. Hong S. W. (2024). Skeletal muscle regeneration with 3D bioprinted hyaluronate/gelatin hydrogels incorporating MXene nanoparticles. Int. J. Biol. Macromol. 265, 130696. 10.1016/j.ijbiomac.2024.130696 38458288

[B51] Judge A. Dodd M. S. (2020). Metabolism. Essays Biochem. 64, 607–647. 10.1042/EBC20190041 32830223 PMC7545035

[B52] Juhas M. Bursac N. (2013). Engineering skeletal muscle repair. Curr. Opin. Biotechnol. 24, 880–886. 10.1016/j.copbio.2013.04.013 23711735 PMC3766474

[B53] Kang M. J. Moon J. W. Lee J. O. Kim J. H. Jung E. J. Kim S. J. (2022). Metformin induces muscle atrophy by transcriptional regulation of myostatin *via* HDAC6 and FoxO3a. J. Cachexia Sarcopenia Muscle 13, 605–620. 10.1002/jcsm.12833 34725961 PMC8818615

[B54] Kang J. Benjamin D. I. Kim S. Salvi J. S. Dhaliwal G. Lam R. (2024). Depletion of SAM leading to loss of heterochromatin drives muscle stem cell ageing. Nat. Metab. 6, 153–168. 10.1038/s42255-023-00955-z 38243132 PMC10976122

[B55] Kangalgil M. Küçük A. O. Ulusoy H. Özçelik A. Ö. (2024). Nutrition determinants of acute skeletal muscle loss in critically ill patients: a prospective observational cohort study. Nutr. Clin. Pract. 39, 579–588. 10.1002/ncp.11086 37877164

[B56] Klumpp D. Horch R. E. Kneser U. Beier J. P. (2010). Engineering skeletal muscle tissue – new perspectives *in vitro* and *in vivo* . J. Cell. Mol. Med. 14, 2622–2629. 10.1111/j.1582-4934.2010.01183.x 21091904 PMC4373482

[B57] Kornegay J. N. (2017). The golden retriever model of Duchenne muscular dystrophy. Skelet. Muscle 7, 9. 10.1186/s13395-017-0124-z 28526070 PMC5438519

[B58] Lee H. Ha T. Y. Jung C. H. Nirmala F. S. Park S.-Y. Huh Y. H. (2021). Mitochondrial dysfunction in skeletal muscle contributes to the development of acute insulin resistance in mice. J. Cachexia Sarcopenia Muscle 12, 1925–1939. 10.1002/jcsm.12794 34605225 PMC8718067

[B59] Lerin C. Goldfine A. B. Boes T. Liu M. Kasif S. Dreyfuss J. M. (2016). Defects in muscle branched-chain amino acid oxidation contribute to impaired lipid metabolism. Mol. Metab. 5, 926–936. 10.1016/j.molmet.2016.08.001 27689005 PMC5034611

[B60] Lian D. Chen M.-M. Wu H. Deng S. Hu X. (2022). The role of oxidative stress in skeletal muscle myogenesis and muscle disease. Antioxidants 11, 755. 10.3390/antiox11040755 35453440 PMC9026549

[B61] Long D. E. Peck B. D. Martz J. L. Tuggle S. C. Bush H. M. McGwin G. (2017). Metformin to Augment Strength Training Effective Response in Seniors (MASTERS): study protocol for a randomized controlled trial. Trials 18, 192. 10.1186/s13063-017-1932-5 28441958 PMC5405504

[B62] Lundsgaard A.-M. Fritzen A. M. Kiens B. (2018). Molecular regulation of Fatty acid oxidation in skeletal muscle during aerobic exercise. Trends Endocrinol. Metab. 29, 18–30. 10.1016/j.tem.2017.10.011 29221849

[B63] Lyu Q. Wen Y. He B. Zhang X. Chen J. Sun Y. (2022). The ameliorating effects of metformin on disarrangement ongoing in gastrocnemius muscle of sarcopenic and obese sarcopenic mice. Biochim. Biophys. Acta BBA - Mol. Basis Dis. 1868, 166508. 10.1016/j.bbadis.2022.166508 35905940

[B64] Madden L. Juhas M. Kraus W. E. Truskey G. A. Bursac N. (2015). Bioengineered human myobundles mimic clinical responses of skeletal muscle to drugs. eLife 4, e04885. 10.7554/eLife.04885 25575180 PMC4337710

[B65] Maffioletti S. M. Sarcar S. Henderson A. B. H. Mannhardt I. Pinton L. Moyle L. A. (2018). Three-Dimensional human iPSC-Derived artificial skeletal muscles model muscular dystrophies and enable multilineage tissue engineering. Cell Rep. 23, 899–908. 10.1016/j.celrep.2018.03.091 29669293 PMC5917451

[B66] Martínez-Reyes I. Chandel N. S. (2020). Mitochondrial TCA cycle metabolites control physiology and disease. Nat. Commun. 11, 102. 10.1038/s41467-019-13668-3 31900386 PMC6941980

[B67] Martínez-Reyes I. Diebold L. P. Kong H. Schieber M. Huang H. Hensley C. T. (2016). TCA cycle and mitochondrial membrane potential are necessary for diverse biological functions. Mol. Cell 61, 199–209. 10.1016/j.molcel.2015.12.002 26725009 PMC4724312

[B68] Mashili F. L. Austin R. L. Deshmukh A. S. Fritz T. Caidahl K. Bergdahl K. (2011). Direct effects of FGF21 on glucose uptake in human skeletal muscle: implications for type 2 diabetes and obesity. Diabetes Metab. Res. Rev. 27, 286–297. 10.1002/dmrr.1177 21309058

[B69] Merz K. E. Thurmond D. C. (2020). “Role of skeletal muscle in insulin resistance and glucose uptake,” in Comprehensive physiology (John Wiley and Sons, Ltd), 785–809. 10.1002/cphy.c190029 PMC8074531 32940941

[B70] Muscaritoli M. Lucia S. Molfino A. Cederholm T. Rossi Fanelli F. (2013). Muscle atrophy in aging and chronic diseases: is it sarcopenia or cachexia? Intern. Emerg. Med. 8, 553–560. 10.1007/s11739-012-0807-8 22773188

[B71] Nair K. S. Schwartz R. G. Welle S. (1992). Leucine as a regulator of whole body and skeletal muscle protein metabolism in humans. Am. J. Physiol.-Endocrinol. Metab. 263, E928–E934. 10.1152/ajpendo.1992.263.5.E928 1443126

[B72] Obata A. Kubota N. Kubota T. Iwamoto M. Sato H. Sakurai Y. (2016). Tofogliflozin improves insulin resistance in skeletal muscle and accelerates lipolysis in adipose tissue in Male mice. Endocrinology 157, 1029–1042. 10.1210/en.2015-1588 26713783

[B73] Peng Y. Du J. Günther S. Guo X. Wang S. Schneider A. (2022). Mechano-signaling *via* Piezo1 prevents activation and p53-mediated senescence of muscle stem cells. Redox Biol. 52, 102309. 10.1016/j.redox.2022.102309 35395625 PMC9005960

[B74] Pepino M. Y. Kuda O. Samovski D. Abumrad N. A. (2014). Structure-Function of CD36 and importance of Fatty acid signal transduction in fat metabolism. Annu. Rev. Nutr. 34, 281–303. 10.1146/annurev-nutr-071812-161220 24850384 PMC4329921

[B75] Petrocelli J. J. Mahmassani Z. S. Fix D. K. Montgomery J. A. Reidy P. T. McKenzie A. I. (2021). Metformin and leucine increase satellite cells and collagen remodeling during disuse and recovery in aged muscle. FASEB J. 35, e21862. 10.1096/fj.202100883R 34416035 PMC8384128

[B76] Phielix E. Mensink M. (2008). Type 2 diabetes mellitus and skeletal muscle metabolic function. Physiol. Behav. 94, 252–258. 10.1016/j.physbeh.2008.01.020 18342897

[B77] Pillon N. J. Bilan P. J. Fink L. N. Klip A. (2013). Cross-talk between skeletal muscle and immune cells: muscle-derived mediators and metabolic implications. Am. J. Physiol.-Endocrinol. Metab. 304, E453–E465. 10.1152/ajpendo.00553.2012 23277185

[B78] Rasmussen B. B. Wolfe R. R. (1999). Regulation of fatty acid oxidation in skeletal muscle. Annu. Rev. Nutr. 19, 463–484. 10.1146/annurev.nutr.19.1.463 10448533

[B79] Rodgers J. T. King K. Y. Brett J. O. Cromie M. J. Charville G. W. Maguire K. K. (2014). mTORC1 controls the adaptive transition of quiescent stem cells from G0 to G(Alert). Nature 510, 393–396. 10.1038/nature13255 24870234 PMC4065227

[B80] Rowan J. A. Rush E. C. Plank L. D. Lu J. Obolonkin V. Coat S. (2018). Metformin in gestational diabetes: the offspring follow-up (MiG TOFU): body composition and metabolic outcomes at 7-9 years of age. BMJ Open Diabetes Res. 6, e000456. 10.1136/bmjdrc-2017-000456 PMC5905785 29682291

[B81] Ryall J. G. Dell’Orso S. Derfoul A. Juan A. Zare H. Feng X. (2015). The NAD+-Dependent SIRT1 deacetylase translates a metabolic switch into regulatory epigenetics in skeletal muscle stem cells. Cell Stem Cell 16, 171–183. 10.1016/j.stem.2014.12.004 25600643 PMC4320668

[B82] Sebastián D. Guitart M. García-Martínez C. Mauvezin C. Orellana-Gavaldà J. M. Serra D. (2009). Novel role of FATP1 in mitochondrial fatty acid oxidation in skeletal muscle cells. J. Lipid Res. 50, 1789–1799. 10.1194/jlr.M800535-JLR200 19429947 PMC2724792

[B83] Seyssel K. Alligier M. Meugnier E. Chanseaume E. Loizon E. Canto C. (2014). Regulation of energy metabolism and mitochondrial function in skeletal muscle during lipid overfeeding in healthy men. J. Clin. Endocrinol. Metab. 99, E1254–E1262. 10.1210/jc.2013-4379 24684464

[B84] Shang M. Cappellesso F. Amorim R. Serneels J. Virga F. Eelen G. (2020). Macrophage-derived glutamine boosts satellite cells and muscle regeneration. Nature 587, 626–631. 10.1038/s41586-020-2857-9 33116312 PMC7116844

[B85] Soro-Arnáiz I. Fitzgerald G. Cherkaoui S. Zhang J. Gilardoni P. Ghosh A. (2024). GLUD1 determines murine muscle stem cell fate by controlling mitochondrial glutamate levels. Dev. Cell 59, 2850–2865.e8. 10.1016/j.devcel.2024.07.015 39121856

[B86] Sreenivasan K. Rodríguez-delaRosa A. Kim J. Mesquita D. Segalés J. Arco P. G.-D. (2021). CHD4 ensures stem cell lineage fidelity during skeletal muscle regeneration. Stem Cell Rep. 16, 2089–2098. 10.1016/j.stemcr.2021.07.022 PMC8452531 34450038

[B87] Sun K.-X. Jiang X.-Y. Li X. Su Y.-J. Wang J.-L. Zhang L. (2021). Deletion of phosphatidylserine flippase β-subunit Tmem30a in satellite cells leads to delayed skeletal muscle regeneration. Zool. Res. 42, 650–659. 10.24272/j.issn.2095-8137.2021.195 34472226 PMC8455468

[B88] Tomlinson D. J. Erskine R. M. Morse C. I. Winwood K. Onambélé-Pearson G. (2016). The impact of obesity on skeletal muscle strength and structure through adolescence to old age. Biogerontology 17, 467–483. 10.1007/s10522-015-9626-4 26667010 PMC4889641

[B89] Tosato M. Marzetti E. Picca A. Calvani R. (2024). “Sarcopenia,” in Geriatric medicine: a person centered evidence based approach. Editors Wasserman M. R. Bakerjian D. Linnebur S. Brangman S. Cesari M. Rosen S. (Cham: Springer International Publishing), 1213–1233. 10.1007/978-3-030-74720-6_116

[B90] Verkerke A. R. P. Ferrara P. J. Lin C.-T. Johnson J. M. Ryan T. E. Maschek J. A. (2019). Phospholipid methylation regulates muscle metabolic rate through Ca2+ transport efficiency. Nat. Metab. 1, 876–885. 10.1038/s42255-019-0111-2 32405618 PMC7218817

[B91] Verpoorten S. Sfyri P. Scully D. Mitchell R. Tzimou A. Mougios V. (2020). Loss of CD36 protects against diet-induced obesity but results in impaired muscle stem cell function, delayed muscle regeneration and hepatic steatosis. Acta Physiol. 228, e13395. 10.1111/apha.13395 31599493

[B92] Viggars M. Nolan A. Sharples A. Stewart C. (2023). “Skeletal muscle satellite cell physiology and function: complimentary *in vitro* and *in vivo* models and methods,” in Neuromuscular assessments of form and function. Editors Atherton P. J. Wilkinson D. J. (New York, NY: Springer US), 243–274. 10.1007/978-1-0716-3315-1_13

[B93] Vishnolia K. K. Martin N. R. W. Player D. J. Spikings E. Lewis M. P. (2020). Zebrafish skeletal muscle cell cultures: monolayer to three-dimensional tissue engineered collagen constructs. 10.1101/2020.12.10.419168

[B94] Watt M. J. Hoy A. J. (2012). Lipid metabolism in skeletal muscle: generation of adaptive and maladaptive intracellular signals for cellular function. Am. J. Physiol.-Endocrinol. Metab. 302, E1315–E1328. 10.1152/ajpendo.00561.2011 22185843

[B95] Wei Y. Chen K. Whaley-Connell A. T. Stump C. S. Ibdah J. A. Sowers J. R. (2008). Skeletal muscle insulin resistance: role of inflammatory cytokines and reactive oxygen species. Am. J. Physiol.-Regul. Integr. Comp. Physiol. 294, R673–R680. 10.1152/ajpregu.00561.2007 18094066

[B96] Werner J.-U. Tödter K. Xu P. Lockhart L. Jähnert M. Gottmann P. (2018). Comparison of Fatty acid and gene profiles in skeletal muscle in normal and obese C57BL/6J mice before and after blunt muscle injury. Front. Physiol. 9, 19. 10.3389/fphys.2018.00019 29441023 PMC5797686

[B97] Winter L. Wittig I. Peeva V. Eggers B. Heidler J. Chevessier F. (2016). Mutant desmin substantially perturbs mitochondrial morphology, function and maintenance in skeletal muscle tissue. Acta Neuropathol. (Berl.) 132, 453–473. 10.1007/s00401-016-1592-7 27393313 PMC4992032

[B98] Yoshiko A. Maeda H. Takahashi H. Koike T. Tanaka N. Akima H. (2022). Importance of skeletal muscle lipid levels for muscle function and physical function in older individuals. Appl. Physiol. Nutr. Metab. 47, 649–658. 10.1139/apnm-2021-0685 35839289

[B99] You J.-S. Singh N. Reyes-Ordonez A. Khanna N. Bao Z. Zhao H. (2021). ARHGEF3 regulates skeletal muscle regeneration and strength through autophagy. Cell Rep. 34, 108594. 10.1016/j.celrep.2020.108594 33406419 PMC10232017

[B100] Yue F. Oprescu S. N. Qiu J. Gu L. Zhang L. Chen J. (2022). Lipid droplet dynamics regulate adult muscle stem cell fate. Cell Rep. 38, 110267. 10.1016/j.celrep.2021.110267 35045287 PMC9127130

[B101] Zammit P. S. Golding J. P. Nagata Y. Hudon V. Partridge T. A. Beauchamp J. R. (2004). Muscle satellite cells adopt divergent fates: a mechanism for self-renewal? J. Cell Biol. 166, 347–357. 10.1083/jcb.200312007 15277541 PMC2172269

[B102] Zhang J. Muri J. Fitzgerald G. Gorski T. Gianni-Barrera R. Masschelein E. (2020). Endothelial lactate controls muscle regeneration from ischemia by inducing M2-like macrophage polarization. Cell Metab. 31, 1136–1153. 10.1016/j.cmet.2020.05.004 32492393 PMC7267778

[B103] Zhou B. Lin W. Long Y. Yang Y. Zhang H. Wu K. (2022). Notch signaling pathway: architecture, disease, and therapeutics. Signal Transduct. Target. Ther. 7, 95–33. 10.1038/s41392-022-00934-y 35332121 PMC8948217

